# *liver-enriched gene 1a* and *1b* Encode Novel Secretory Proteins Essential for Normal Liver Development in Zebrafish

**DOI:** 10.1371/journal.pone.0022910

**Published:** 2011-08-09

**Authors:** Changqing Chang, Minjie Hu, Zhihui Zhu, Li Jan Lo, Jun Chen, Jinrong Peng

**Affiliations:** 1 College of Animal Sciences, Zhejiang University, Hangzhou, People's Republic of China; 2 College of Life Sciences, Zhejiang University, Hangzhou, People's Republic of China; Institute of Zoology, Chinese Academy of Sciences, China

## Abstract

*liver-enriched gene 1* (*leg1*) is a liver-enriched gene in zebrafish and encodes a novel protein. Our preliminary data suggested that Leg1 is probably involved in early liver development. However, no detailed characterization of Leg1 has been reported thus far. We undertook both bioinformatic and experimental approaches to study *leg1* gene structure and its role in early liver development. We found that Leg1 identifies a new conserved protein superfamily featured by the presence of domain of unknown function 781 (DUF781). There are two copies of *leg1* in zebrafish, namely *leg1a* and *leg1b*. Both *leg1a* and *leg1b* are expressed in the larvae and adult liver with *leg1a* being the predominant form. Knockdown of Leg1a or Leg1b by their respective morpholinos specifically targeting their 5′-UTR each resulted in a small liver phenotype, demonstrating that both Leg1a and Leg1b are important for early liver development. Meanwhile, we found that injection of leg1-ATG^MO^, a morpholino which can simultaneously block the translation of Leg1a and Leg1b, caused not only a small liver phenotype but hypoplastic exocrine pancreas and intestinal tube as well. Further examination of leg1-ATG^MO^ morphants with early endoderm markers and early hepatic markers revealed that although depletion of total Leg1 does not alter the hepatic and pancreatic fate of the endoderm cells, it leads to cell cycle arrest that results in growth retardation of liver, exocrine pancreas and intestine. Finally, we proved that Leg1 is a secretory protein. This intrigued us to propose that Leg1 might act as a novel secreted regulator that is essential for liver and other digestive organ development in zebrafish.

## Introduction

Liver expresses vast varieties of genes, including liver-specific and/or –enriched genes, to encode different proteins necessary for executing its diverse functions [1]–[3]. For example, liver produces and secretes a variety of serum proteins, such as albumin, fibrinogen, prothrombin and antithrombin etc, to maintain homeostasis of the body [4]. In many cases, the expression of liver-specific and/or –enriched genes are under the control of a network formed by transcription factors including hepatic nuclear factors HNF1, HNF3, HNF4, HNF6, and C/EBPα (CCAAT/enhancer binding protein) etc [3]–[6]. Extensive genetic studies have demonstrated that, in addition to their roles in controlling the expression of metabolic genes, all HNF proteins are also essential for liver organogenesis.

The process of liver organogenesis is governed by a network formed by HNF factors, GATA factors, and morphogens including FGF, BMP and Wnt2 [7]–[9]. This genetic network coordinates expression and functions of many genes to guide the liver to develop into the right size and shape at the right time and place. Much has been learned about the physiological and biochemical functions of genes expressed in the adult liver [3], [10]–[12]. However, due to restriction of experimental systems, few studies have been carried to identify genes with their expression enriched in both a developing liver and a mature liver and thereof their functions in both processes. This work is particularly important since continuous expression of this set of live-enriched genes from the fetal to adult stages suggest their essential roles in both early liver development and stem cell function and/or the status maintainence in an adult liver [13]. The latter function is crucial for liver regeneration after hepatectomy.

Zebrafish (*Denrio rerio*) has been proven to be an excellent genetic model system to study both processes of liver development and liver regeneration [9], [14], [15]. Liver organogenesis in zebrafish shares similar mechanism with mammals and other vertebrates with regard to the processes of morphogenesis and molecular control [16]–[20]. In addition, a number of novel positive/negative factors essential for liver organogenesis in zebrafish, including Vps18, Sox9a and Fgr (Foie gras), Npo (Nil per os), Def etc [21]–[24], have been identified via studying genetic mutants.

We previously reported the identification of 129 adult liver-enriched genes in zebrafish through the microarray approach [3]. Further whole-mount *in situ* hybridization (WISH) studies revealed that 69 out of these 129 genes were also enriched in the embryonic liver [3]. Our main interest is in understanding if such adult liver-enriched genes also function in early liver development. A novel gene, *leg1* (*live-enriched gene 1*) was among these 69 liver-enriched genes. Our preliminary functional study via morpholino-mediated gene knock-down approach showed that that *leg1* morphant conferred a small liver phenotype [3]. In this work, we reported our more detailed studies on the *leg1* gene. We found that there are two copies of *leg1*, namely *leg1a* and *leg1b*, in zebrafish, and Leg1 proteins are highly conserved among vertebrates. We confirmed that both Leg1a and Leg1b play important roles during liver development. More importantly, we demonstrated that Leg1 is a secretory protein. These results suggest that Leg1 might function as a novel secreted regulator for the liver development.

## Methods

### Ethics Statement

This study does not involve non-human primates. Research work has been performed in full accordance to the requirement by ‘Governing Regulation for the Use of Experimental Animals in Zhejiang Province’ (Zhejiang Provincial Government Order No 263, released in August 27, 2009, effective from October 1, 2010). According to the Chapter for Biosafety and Animal Ethics (Chapter 4), as stated in Line 28: ‘Units and individuals who are conducting the production and use of experimental animal production, should treat animals humanely and protect animal welfare, should not tease and abuse animals. The use of experimental animals should be in accordance to the scientific, rational and humane requirements. It is advised and encouraged to minimize the use of laboratory animals to reduce suffering of animals to be disposed of, and to explore of alternative methods in replacing animal testing and use’, ethical approval is not stated to be required for scientific research using adult or embryonic zebrafish by the Regulation. Every effort was made to minimize any suffering of the animals used in this study. Zebrafish (*Danio rerio*) wild type AB strain was used in this study. Adult zebrafish was euthanized in Tricane solution before being dissected for tissue collection. Zebrafish was raised up and maintained in the standard Zebrafish Unit (produced by Aisheng Zebrafish Facility Manufacturer Company, Beijing, China).

### Whole-mount in situ hybridization (WISH)

RNA probes were obtained from *fabp10a* and *leg1a* plasmids respectively by in vitro transcription (T3 RNA polymerase kit, Promega), and were labeled with digoxigenin-UTP (DIG-labeling) (Roche Diagnostics). WISH was performed as described previously [3].

### Zebrafish Leg1 monoclonal antibody preparation

*leg1a* cDNA full length sequence was amplified using primers leg1a-fd and leg1a-re (leg1a-fd, forward: 5′-TGTCTGGATTCGGTTTCCTGCGATCAGTG-3′; leg1a-re, reverse: 5′-TACCACGAATTCAGCAGCTGGTGGACATCT-3′) and cloned into pGEX-6P-1 (Clontech) between *BamHI* and *EcoRI* cloning sites. Leg1 protein was expressed in *E. coli*, purified and used as the antigen. Monoclonal antibody against zebrafish Leg1 was prepared by the Monoclonal Antibody Unit in the Institute of Molecular and Cell Biology, Singapore.

### RNA and protein analysis

Total RNA was extracted from embryos at different developmental stages using TRIzol (Invitrogen, USA). Probes were DIG-labeled and Northern blotting was performed accordingly to manufacturer's instruction (Roche Diagnostics) [3]. For analyzing *leg1a* and *leg1b* expression ratio, first strand cDNA was synthesized using Superscript II (Invitrogen, USA) followed by PCR with forward primer (Fwd 5′-GCCCCGGGGAGCAGGAGAATC-3′) and reverse primer (Rev 5′-GCTGGACCCGGGGAACTTTG-3′). PCR products were cloned into pGEM-Teasy vector (Promega). 65 clones from each developmental stage were randomly picked and sequenced. Sequences obtained were analyzed to identify the ones corresponding to *leg1a* or *leg1b*. For western blotting, total protein was extracted using standard SDS sample buffer. Western blotting was performed as described previously [25] using monoclonal antibody against zebrafish Leg1 as the primary antibody. Total 12 adult zebrafish (five months old) were used extracting total RNA and protein, respectively.

### Morpholinos

Morpholinos were obtained from GeneTools (Philomath, USA). leg1-MO^ATG^ morpholino (5′-CCATCTCAGACATCTAGCAGGACTG-3′) was designed to target the translation start site regions of both *leg1a* and *leg1b*. Its 5-base mismatch morpholino (5′-CCATgTCAcACATgTAGCAcGAgTG-3′) was designed and used as the mismatch control (st-MO). leg1a-MO (5′-AGTCCAGCAGAGAGGAGCTTTAATC-3′) and leg1b-MO (5′-CCGGGCCACATACTGAATGGAATGA-3′) morpholinos were designed to target the 5′-UTRs of *leg1a* and *leg1b*, respectively. 1 nl of leg1-MO^ATG^ (0.5 nmol/µl), st-MO control (0.5 nmol/µl), leg1a-MO (0.2 nmol/µl), or leg1b-MO^UTR^ (0.7 nmol/µl) was injected into one-cell stage embryos.

### mRNA rescue

*leg1a* and *leg1b* cDNAs were obtained via RT-PCR using primer pairs shared by *leg1a* and *leg1b* (leg1-fd1, forward: 5′-TCAGGAATTCGATGGGTTTCCTGCGATCAG-3′, leg1-re1, reverse: 5′-TCAGTTCTAGATCAGCAGCTGGTGGACAT-3′) and were cloned to pCS2+ vector by *EcoRI* and *XbaI* cloning sites. The identity of *leg1a* or *leg1b* was determined based on SNPs (single nucleotide polymorphism) between these two homologs after sequencing individual clones. *leg1a* and *leg1b* mRNAs were obtained from their respective plasmids via *in vitro* transcription using the Message Machine Kit (Ambion). For morphant phenotype rescue, 0.3 ng of *in vitro* transcribed WT *leg1a* or *leg1b* mRNA was injected into one-cell stage embryos.

### 5′ and 3′ RACE

The FirstChoice® RLM-RACE kit (Ambion) was used to determine the transcription start sites of *leg1* and *leg1b*, and to obtain the 5′-UTR and 3′-UTR sequences corresponding to *leg1a* and *leg1b*, respectively. Experiments were carried out according to the manufacturer's instruction with total RNA as the starting material for first-strand cDNA synthesis.

### Phosphorylated Histone 3 (PH3) immuostaining and TUNEL assay

Embryos were collected at 38 hpf and 3 dpf, respectively. Embryos were fixed and embedded and sectioned as described [22]. Cell cycle progression was analyzed by immunostaining PH3 and cell apoptosis by TUNEL as described [22].

### Liver size measurement

The liver of an embryo at 90 hpf was marked out by WISH using the *fabp10a* probe. We assumed that the thicker part of the liver would be stained darker hence blocking more light penetration. This in turn will yield stronger signal intensity upon negative image capturing. Therefore, we could use signal intensities to infer the liver size. Firstly, an image of the liver was captured from the left lateral view after aligning two eyes of the embryo vertically so that to avoid the positional discrepancy among each individual embryo. Next, the image of individual embryo was used to obtain *fabp10a* staining signal intensity by Nikon image system (NIS-elements D v3.0).

### Phylogenetic analysis

Leg1 homologs from different species were selected from the cluster of proteins containing the DUF781 domain identified using PANTHER (Protein Analysis Through Evolutionary Relationships, http://www.pantherdb.org/). Protein sequences were then retrieved and aligned via Clustal W program. Phylogenetic tree was built with MEGA 4 program [26].

### Signal peptide prediction

HMM (Hidden Markov models) method in SignalP (http://www.cbs.dtu.dk/services/SignalP/) was used to predict signal peptide in Leg1. The probabilities that the Leg1 leading sequence is a signal peptide and signal anchor are 0.995 and 0.004, respectively. And the probability to be a signal peptidase cleavage site, locating between position 22 and 23, is 0.924 [27].

## Results

### *leg1a* and *leg1b* are two closely linked homologous genes in zebrafish

**Figure 1:**
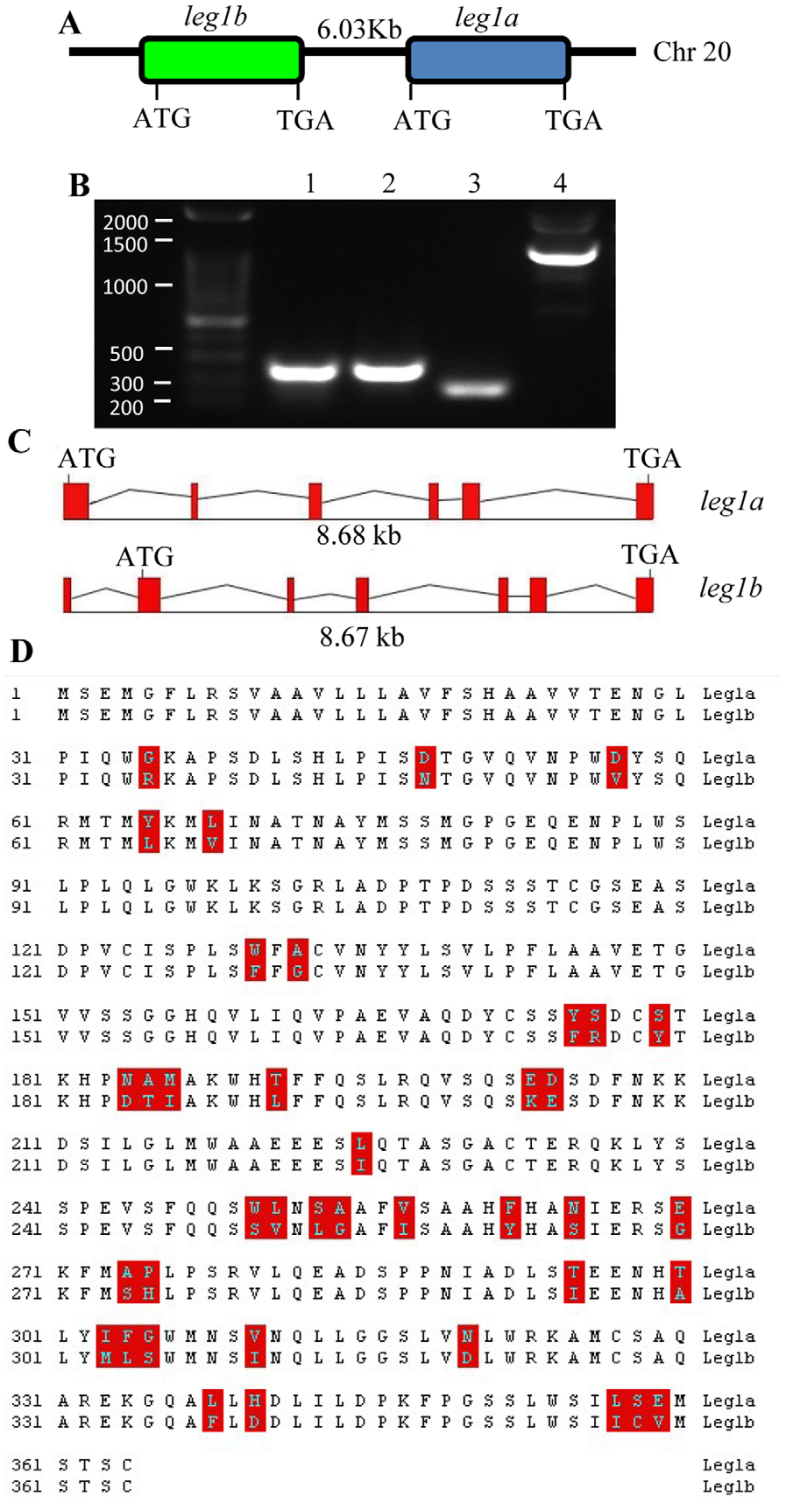
Leg1a and Leg1b are two closely related homologs in zebrafish. (A) Diagram showing the arrangement of *leg1a* and *leg1b* on chromosome 20. (B) RT-PCR showing the expression of *leg1a* and *leg1b* in adult liver. Primer pairs amplifying *leg1a* are derived from exon1 of *leg1a* (lanes 1 and 2), *leg1b* are from exon1 and exon2 of *leg1b* (lanes 3 and 4). Lanes 1 and 3: cDNA as template; lanes 2 and 4: genomics DNA as template. (C) Diagram showing *leg1a* and *leg1b* genomic structures. Red box: exon; uneven line: introns. (D) Alignment of Leg1a and Leg1b amino acid sequences to highlight the 39 different amino acids between them (boxed in red).

We previously reported a brief data showing that knockdown of Leg1 expression by a morpholino (leg1-MO^ATG^) targeting the start codon ATG of *leg1* caused smaller liver in zebrafish [3]. Notably, there is no other report on Leg1 in any systems thus far. We initiated the work with the purpose to characterize zebrafish *leg1* gene in more detail. We first blasted the obtained *leg1* cDNA sequence [3] against the zebrafish genome sequence assembled in Zv6 (http://www.sanger.ac.uk/Projects/D_rerio). We found that there is a *leg1* homolog positioned in adjacent to the initial *leg1* gene on chromosome 20 separated by 6.03 kb (counting from the last base of 3′-UTR of *leg1b* to the transcription start site of *leg1a*) (Fig. 1A). To find out whether the newly discovered homolog gene is transcribed, we designed primers for the new homolog based on its genomic sequence for RT-PCR. The RT-PCR products (Fig. 1B) were sequenced and the sequence was found to match the predicted transcript for the new homolog (Fig. S1). Thereafter, these two homologs are designated as *leg1a* and *leg1b*, corresponding to the initial *leg1* and new homolog, respectively. Alignment of *leg1a* and *leg1b* transcribed sequences with their respective genomic DNA sequence reveals that *leg1a* has 6 exons and 5 introns whereas *leg1b* has 7 exons and 6 introns (Fig. 1C). Despite the differences in their genomic structures, both of the coding sequences of *leg1a* and *leg1b* are 1083 nucleotides in length sharing high homology (95.2%) (Fig. S1). Amino acids alignment shows that Leg1a and Leg1b share 90.6% identity and differ only in 39 amino acids (Fig. 1D).

### Phylogenetic analysis of Leg1

**Figure 2:**
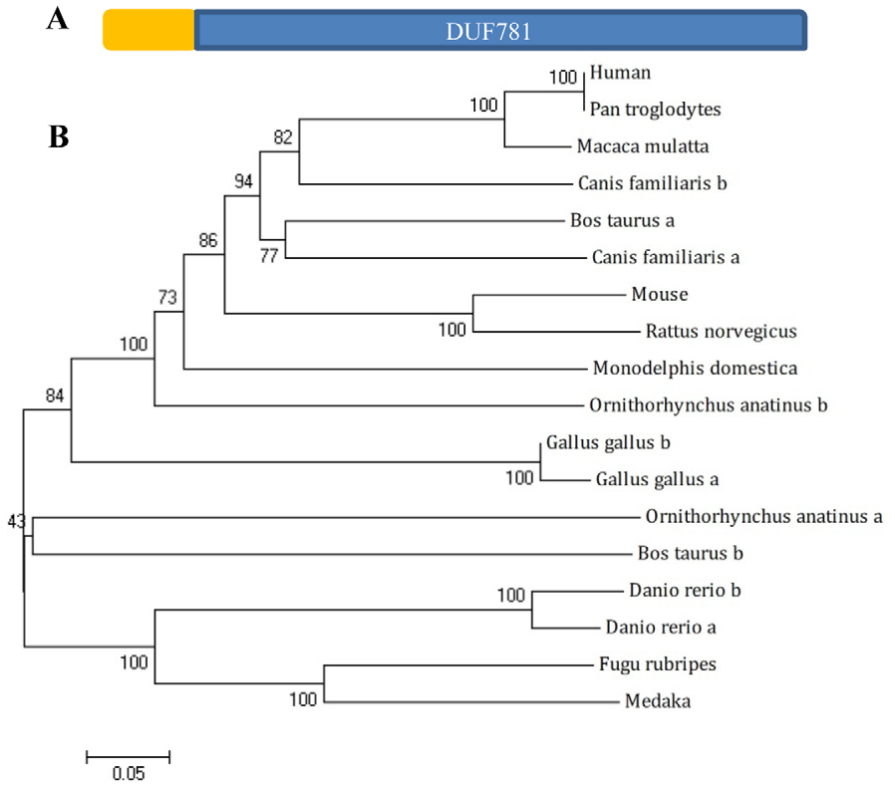
Phylogenetic analysis of Leg1. (A) Leg1 family is featured by the domain of unknown function 781 (DUF781) (blue bar). Yellow bar: N-terminal leading peptide. (B) Phylogenetic analysis of Leg1. Human: NP_001010905.1; *Pan troglodytes*: XP_518733.2; *Macaca mulatta*: NP_001181247.1; *Canis familiaris* a: XP_855514.1; *Canis familiaris* b: XP_864054.1; *Bos taurus* a: ENSBTAG00000017166; *Bos taurus* b: ENSBTAG00000026578; mouse: NP_080612.1; *Rattus norvegicus*: XP_001059712.1; *Monodelphis domestica*: XP_001380262.1; *Ornithorhynchus anatinus* a: ENSOANG00000002104; *Ornithorhynchus anatinus* b: ENSOANG00000008387; *Gallus gallus* a: XP_419749; *Gallus gallus* b: XP_001232481; *Danio rerio* a: NP_001093526.1; *Danio rerio* b: NP_998368.1; *Fugu rubripes*: ENSTRUT00000034344; medaka: ENSORLT00000022610.

Extensive database search reveals that Leg1a and Leg1b represent a new family of proteins present in other animal species characterized by a conserved domain DUF781 (domain of unknown function) (Fig. 2A). Phylogenetic analysis shows that Leg1 is well-conserved among vertebrates. Zebrafish Leg1a and Leg1b are closely related to Leg1 homologs in teleost including *fugu rubripes* (49% identity) and medaka fish (50% identity) but display a significant evolutional distance from mammals including rat (36% identity), mouse (36% identity), dog (34% identity), Rhesus monkey (36% identity) and human (36% identity) (Fig. 2B).

### *leg1a* and *leg1b* are differentially expressed

**Figure 3:**
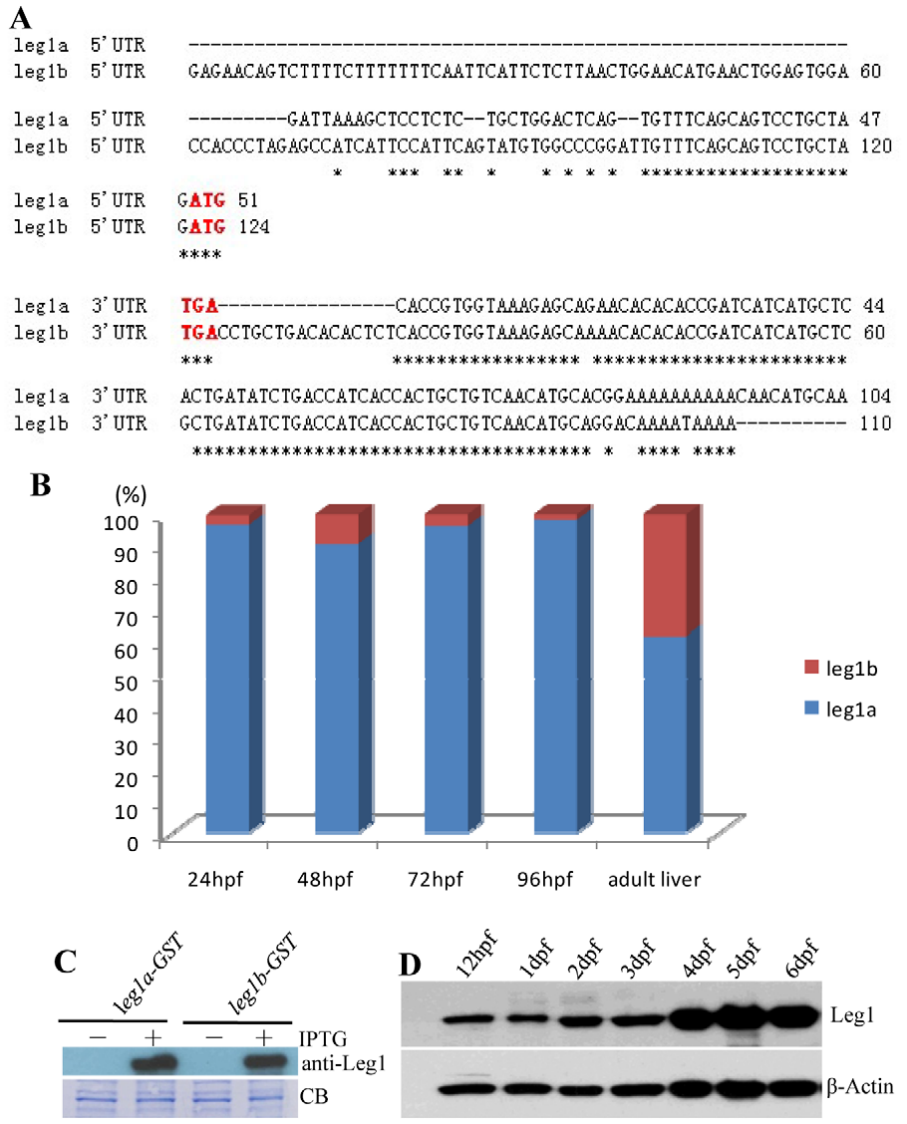
*leg1a* and *leg1b* are differentially expressed. (A) Alignment of 5′- and 3′-UTRs of *leg1a* and *leg1b* transcripts, respectively. Asterisk highlights identical bases. Translation start codon ATG and stop codon TGA are lettered in red. (B) Comparison of *leg1a* and *leg1b* expression at different developmental stages as indicated. Data are presented in percentage (blue bar: *leg1a*; red bar: *leg1b*). (C) Anti-Leg1 monoclonal antibody recognizes both Leg1a and Leg1b which were induced to express in *E. coli* by isopropyl β-D-1-thiogalactopyranoside (IPTG). (D) Western blotting analysis of total Leg1 (Leg1a+Leg1b) expression in embryos at stages as indicated.

The facts that *leg1a* and *leg1b* share high sequence homology in the coding region and are neighboring to each other on chromosome 20 prompt us to investigate whether *leg1a* and *leg1b* are equally expressed in zebrafish at different developmental stages. We first performed 5′- and 3′-RACE to identify the 5′- and 3′-untranslated regions (UTR) for *leg1a* and *leg1b*, respectively. Sequence alignment revealed that, in addition to the high homology shared in the coding region, the 3′-UTRs of *leg1a* and *leg1b* are also highly homologous except that *leg1b* has additional 16 nucleotides just after the stop codon TGA (Fig. 3A). On the other hand, the 5′-UTRs of *leg1a* and *leg1b* are totally divergent except that they share 20 identical nucleotides just ahead of the start codon ATG (Fig. 3A).

The high homology between *leg1a* and *leg1b* makes it difficult to distinguish their expression patterns based on WISH or northern blotting using probes derived from their coding regions. To address this problem, we performed RT-PCR using a pair of primers perfectly matching both *leg1a* and *leg1b* sequences and cloned RT-PCR products into the T-vector. Bacteria colonies were randomly picked and sequenced to identify clones corresponding to *leg1a* or *leg1b* based on SNPs (single nucleotide polymorphism) between *leg1a* and *leg1b*. The result showed that, for embryos examined at 1dpf, 2 dpf, 3 dpf, and 4 dpf, the percentage of clones representing *leg1a* was approximately 97%, 90%, 96%, 98% and was accordingly higher than that for *leg1b* (approximately 3%, 10%, 4%, 2%), (Fig. 3B), demonstrating that *leg1a* transcripts are more abundant than *leg1b* transcripts during embryogenesis. We also checked *leg1a* and *leg1b* expression in the adult liver and found that *leg1a* was also more dominantly expressed (61%) than was *leg1b* (39%) (Fig. 3B).

The above data suggest that *leg1a* and *leg1b* expression are differentially regulated. Gene expression is controlled and regulated by its promoter. We retrieved 3 kb of genomic DNA sequences upstream of *leg1a* and *leg1b* transcription start site, respectively, and aligned them using Ebi Tool needle program (Fig. S2). Comparison of *leg1a* and *leg1b* promoter sequences identified two highly conserved regions. The first region spans ∼510 bp (−636 to −1150 for *leg1a*, −2510 to −3016 for *leg1b*, counting from the first base upstream of the transcription start site for *leg1a* and *leg1b*, respectively) (Fig. S2, letters in red). The second region spans ∼310 bp (−1151 to −1468 for *leg1a*, −3068 to −3380 for leg1b, respectively) (Fig. S2, letters in purple). However, all of the rest sequences, especially the 600 bp proximal promoter directly upstream of the transcription start sites, are highly divergent between these two genes (Fig. S2). This might explain the expression difference between leg1a and leg1b at different developmental stages.

### Both Leg1a and Leg1b are important for normal liver development

A monoclonal antibody recognizing both Leg1a and Leg1b (Fig. 3D) was used to analyze the temporal expression patterns of total Leg1 (Leg1a+Leg1b). The results showed that total Leg1 can be detected in the larvae from 12 hpf onwards with a gradual increase in signal intensity until 6 dpf (Fig. 3E).

**Figure 4:**
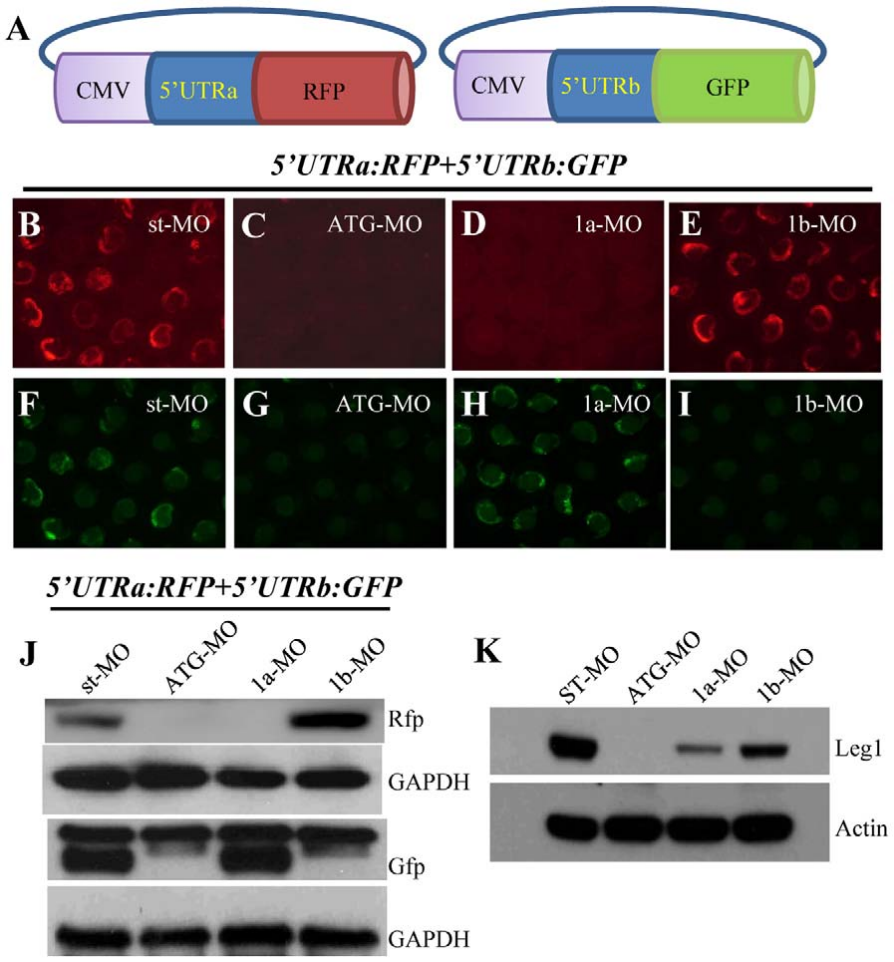
Knockdown of Leg1a or Leg1b protein expression with their specific morpholinos. (A) Diagram showing construction of *leg1a-5′-UTR:rfp* (*5′UTRa:RFP*) and *leg1b-5′-UTR:gfp* (*5′UTRb:GFP*) plasmids. (B–I) *5′UTRa:RFP* and *5′UTRb:GFP* were mixed and co-injected with st-MO (B, F), leg1-MO^ATG^ (C, G) or leg1a-MO (D, H), or leg1b-MO (E, I). Rfp (B–E) and Gfp (F–I) fluorescence was visualized under a Nikon fluorescence microscope. (J) Western blotting analysis of Rfp and Gfp proteins in the injected embryos as indicated using antibodies against Rfp and Gfp, respectively. (K) Western blotting analysis of total Leg1 in different morphants as indicated.

We reported previously that leg1-MO^ATG^ injection led to a small liver phenotype [3]. Rechecking the morpholino sequence we noticed that leg1-MO^ATG^ in fact, because of sequence conservation between *leg1a* and *leg1b*, targeted both *leg1a* and *leg1b* at their respective translation start site regions (leg1-MO^ATG^). Therefore, the morphant phenotype observed [3] was believed to be due to knockdown of both Leg1a and Leg1b in the morphants. To prove this speculation, we checked total Leg1 proteins in leg1-MO^ATG^ morphants and found that total Leg1 protein expression was almost depleted in the protein extracts from the leg1-MO^ATG^ morphants at 3dpf (Fig. 4K).

To determine which homolog(s), Leg1a or Leg1b or both, play(s) a role in liver development, we designed two morpholinos, leg1a-MO and leg1b-MO, specifically targeting the 5′-UTR of *leg1a* and *leg1b*, respectively. To check the potency of these two mopholinos, the 5′-UTR sequences of *leg1a* and *leg1b* was cloned to the upstream of the reporter gene *rfp* (*red fluorescence protein*) (*leg1a-5′-UTR:rfp*) and *gfp* (*green fluorescence protein*) (*leg1b-5′-UTR:gfp*), respectively (Fig. 4A). The *leg1a-5′-UTR:rfp* and *leg1b-5′-UTR:gfp* plasmid DNA were mixed and co-injected with leg1-MO^ATG^, or leg1a-MO, or leg1b-MO into embryos at one-cell stage. At 10 hpf, Rfp or Gfp fluorescence was observed under a fluorescence microscope. Meanwhile, total proteins were extracted and subjected to western analysis. As expected, we found that leg1-MO^ATG^ blocked both Rfp and Gfp expression (Fig. 4B, C, F, G, J). On the other hand, leg1a-MO but not leg1b-MO diminished the expression of Rfp fluorescence (Fig. 4D, H, J) and *vice versa*, leg1b-MO but not leg1a-MO abrogated the expression of Gfp fluorescence in the injected embryos (Fig. 4E, I, J). These data demonstrate that these two morpholinos worked with high specificity and efficiency.

**Figure 5:**
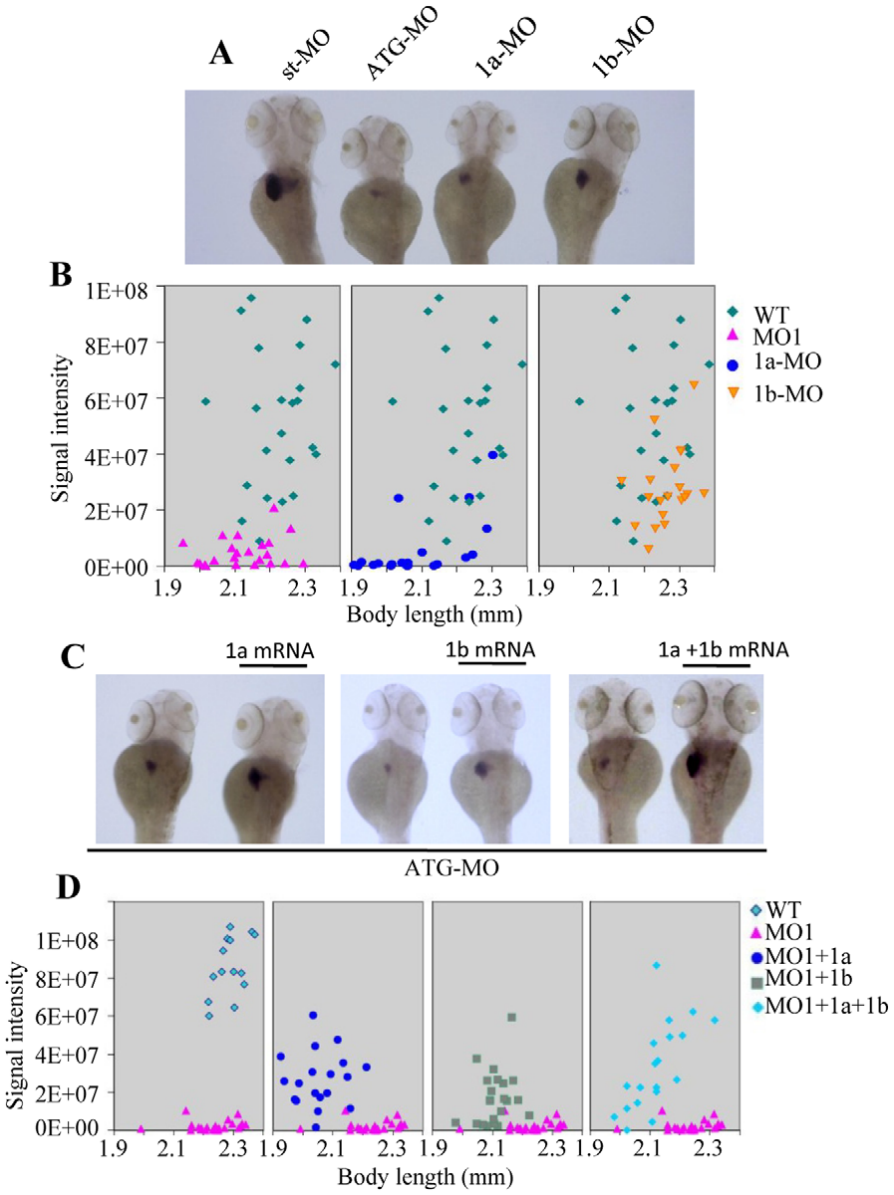
Both Leg1a and Leg1b are essential for normal liver development. (A) leg1-MO^ATG^ (ATG-MO), leg1a-MO (1a-MO) and leg1b-MO (1b-MO) all resulted in small liver phenotype but with different severity. (B) Statistical analysis of liver size in leg1-MO^ATG^ (ATG-MO), leg1a-MO (1a-MO) or leg1b-MO (1b-MO) morphants based on WISH signal intensity of *fabp10a*. Signal intensity (Y-axis) was plotted against body length (X-axis). 20–23 embryos were used for statistical analysis in each case. (C) WISH using *fabp10a* probe to examine rescue of the small liver phenotype caused by leg1-MO^ATG^ with *leg1a* (1a mRNA), *leg1b* (1b mRNA) mRNA, or combination of *leg1a* and *leg1b* mRNA (1a+1b mRNA). (D) Statistical analysis of liver size in each individual leg1-MO^ATG^ morphant based on WISH signal intensity of *fabp10a*. 19–23 embryos were used for statistical analysis in each case except for WT (14 embryos were used).

Next, we compared the efficiencies of leg1a-MO and leg1b-MO on the knockdown of total endogenous Leg1 protein expression. While leg1-MO^ATG^ depleted almost all total Leg1 protein expression leg1a-MO was found to deplete more than 75% of total Leg1. On the other hand, leg1b-MO depleted approximately half of total Leg1 protein (Fig. 4K). Examination of liver development in leg1a-MO and leg1b-MO morphants using the liver specific marker *fabp10a* probe revealed that both morpholinos resulted in liver size reduction. However, leg1a-MO caused a more severe small liver phenotype than did leg1b-MO (Fig. 5A, B). In fact, leg1a-MO is near as potent as leg1-MO^ATG^ regarding their effects on liver development (Fig. 5B). Together, these results suggest that both Leg1a and Leg1b are necessary for normal liver development.

### Leg1a and Leg1b function partially redundant during liver development

Since Leg1a and Leg1b share high homology with each other, it is reasonable to speculate that the functions of these two proteins are probably fully redundant and the degree of severity of small liver exhibited by the leg1a-MO and leg1b-MO morphants would only correlate to Leg1a and Leg1b expression levels in the liver. This appeared to be the case since leg1a-MO morphants displayed more severe phenotype than did leg1b-MO (Fig. 5A, B) when considering *leg1a* is expressed higher than is *leg1b* in the liver (Fig. 2B). We reasoned if Leg1a and Leg1b are fully functionally redundant, overexpressing either *leg1a* or *leg1b* alone by mRNA injection would then result in rescue of the small liver phenotype conferred by the leg1-MO^ATG^ morphant similar to that by injection of *leg1a* and *leg1b* mRNA combination. Using the marker *fabp10a* to examine injected embryos at 4dpf (Fig. 5C), we found that overexpressing Leg1a or Leg1b alone partially rescued the liver size to a similar extent based on quantifying positive *fabp10a* signal intensity at the liver site in leg1-MO^ATG^ morphants (Fig. 5C, D). However, Leg1a achieved a higher rescue rate (16 out of 20 embryos examined) than did Leg1b (13 out of 23 embryos examined) (Fig. 5D). Whereas co-injection of *leg1a* and *leg1b* mRNA resulted in a more significant recovery of the liver size (15 out of 19 embryos examined) (Fig. 5C, D). These data suggest that functions of Leg1a and Leg1b in liver development are partially redundant.

### Depletion of Leg1 blocks liver expansion but not liver initiation

**Figure 6:**
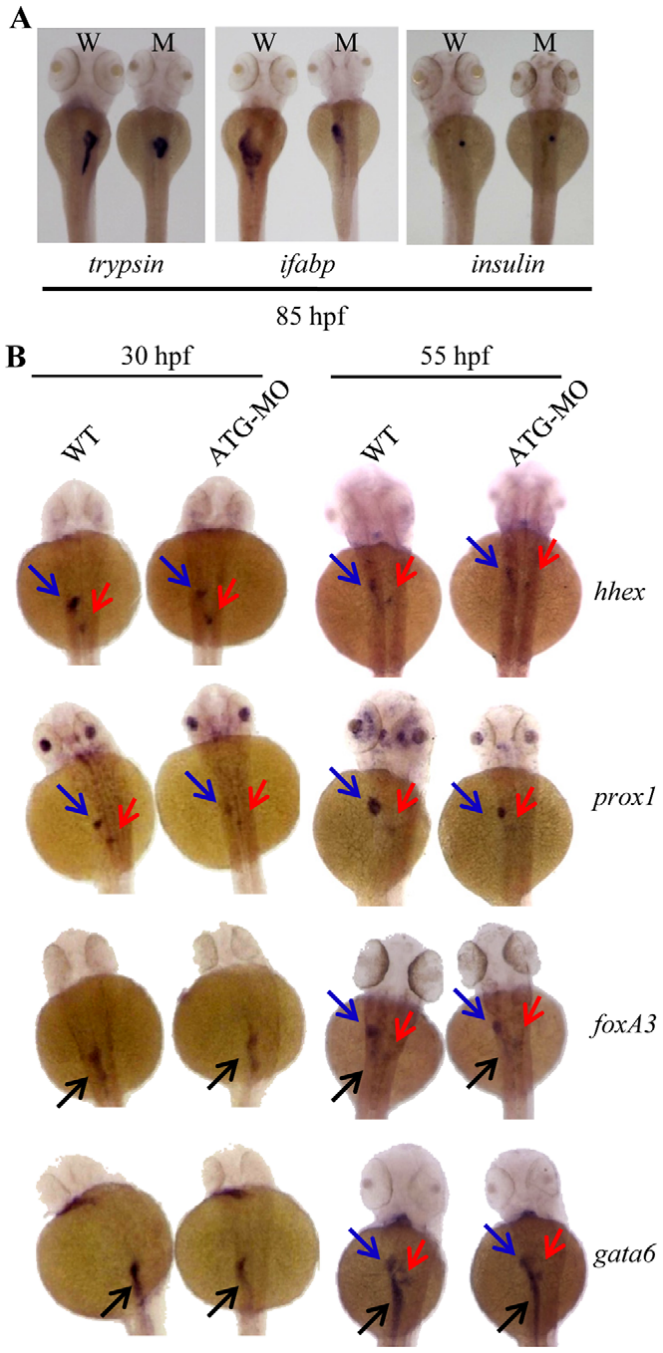
Depletion of Leg1 affects the expansion but not initiation of the liver, exocrine pancreas and intestinal tub. (A) WISH using *trypsin*, *ifabp* and *insulin* as probes to examine the development of exocrine pancreas (trypsin), intestine (ifabp) and endocrine pancreas (insulin) in WT (W) and the leg1-MO^ATG^ morphant (M) embryos at 85 hpf. (B) WISH using early hepatic markers *hhex* and *prox1* and early endoderm markers *foxa3* and *gata6* on WT and leg1-MO^ATG^ morphant (ATG-MO) embryos at 30 hps and 55 hpf, respectively. Blue arrow: liver; red arrow: pancreas; black arrow: intestine.

Digestive organs, including liver, pancreas and intestine, are all originated from the endoderm. We asked if depletion of total Leg1 by leg1-MO^ATG^ morpholino would affect the organogenesis of other digestive organs in addition to the liver. To address this question, WISH was performed on leg1-MO^ATG^ morphants at 85 hpf using exocrine pancreatic marker *trypsin*, intestinal marker *ifabp* (*intestine fatty acid binding protein*) and endocrine pancreas marker *insulin*, respectively. The results showed that the exocrine pancreas tail failed to extend and the intestinal tube was shortened and thinned in the morphant embryos (Fig. 6A). On the other hand, the endocrine pancreas marked by insulin was not obviously affected (Fig. 6A). The data obtained suggest that Leg1 is also essential for organogenesis of exocrine pancreas and intestinal tube.

In zebrafish, the endoderm cells form a rod at around 24 hpf. At around 30 hpf, the endoderm tube undergoes a process termed as ‘gut looping’ which pushes the liver bud to the left while turning the pancreatic bud to the right of the body. At around 55 hpf, the first phase of organogenesis of digestive organs is accomplished [16], [17], [20], [28]. To determine when the inhibitory effect of depletion of Leg1 on liver and other digestive organ development becomes discernable, we performed WISH using early endoderm markers *foxa3* and *gata6* and early hepatic markers *hhex* and *prox1* on embryos at 30 hpf and 55 hpf, respectively. We found that all four markers are expressed in the leg1-MO^ATG^ morphant embryos in a similar pattern as that observed in the wild type (WT) control (Fig. 6B). Carefully examining the hybridization signals of these markers we noticed that, however, expression of all markers was reduced in signal intensity in the leg1-MO^ATG^ morphant embryos (Fig. 6B). This data suggests that depletion of Leg1 has no effect on the determination of the hepatic and pancreatic fate of endoderm cells instead it blocks the expansion growth of these organs.

### Depletion of Leg1 results in cell cycle arrest that leads to small liver phenotype in leg1-MO^ATG^ morphants

**Figure 7:**
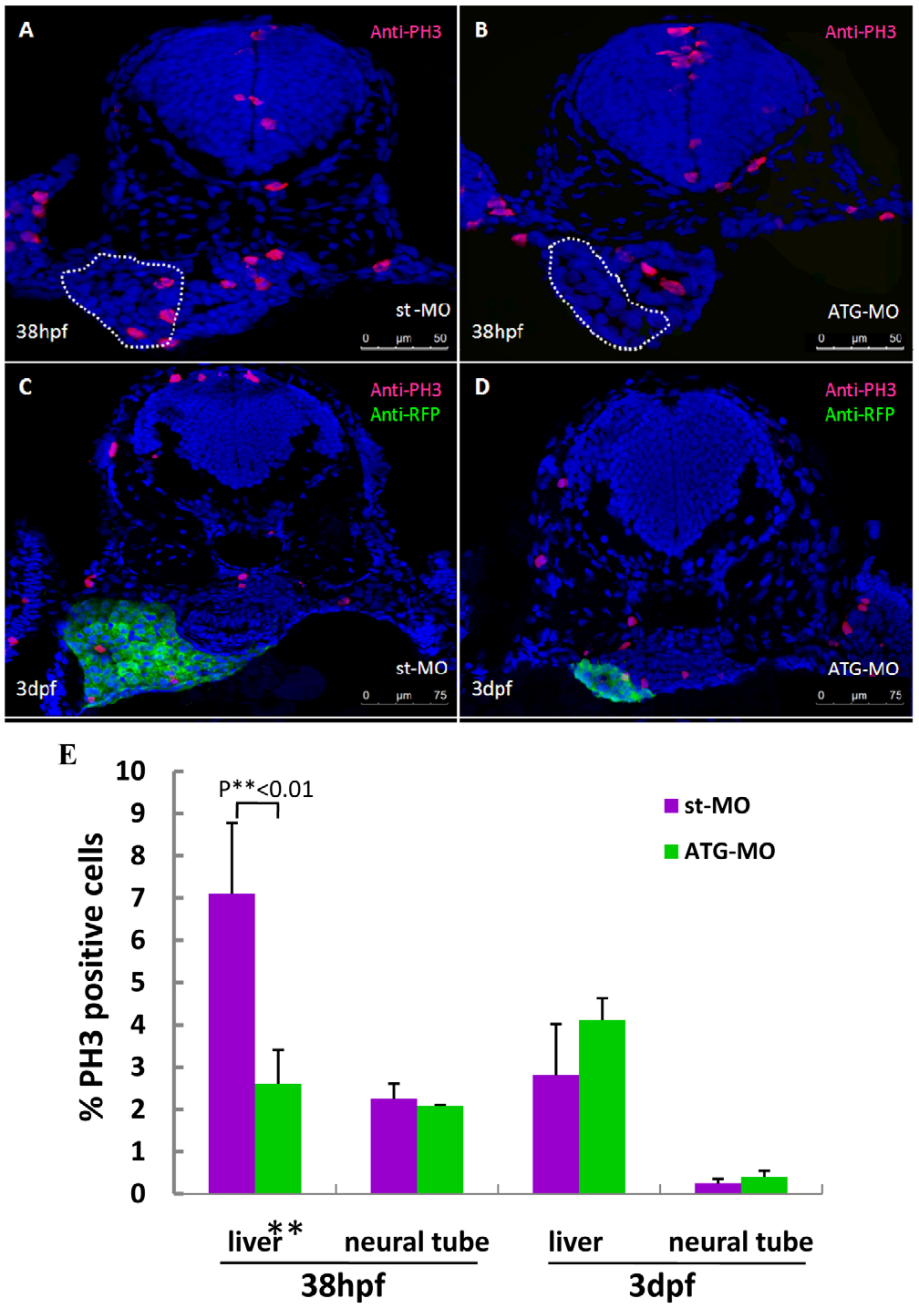
Hypoplastic liver in leg1-MO^ATG^ morphants is caused by cell cycle arrest during the liver budding stage. (A, B) Immunostaining using an anti-PH3 antibody on st-MO (A) and ATG-MO (B) morphants at 38hpf. Liver primordium is circled with a white dotted line. (C, D) Immunostaining using antibodies respectively against PH3 (red) and RFP (green) on cross-sections from st-MO (C) and ATG-MO (D) morphants in the *Tg(lfabp: DsRed; elaA: EGFP)* background at 3dpf. (E) Statistical analysis revealed that the ratio of PH3-positive cells was significantly reduced in the liver primordium of ATG-MO morphants at 38 hpf, but no significant difference in the ratio of PH3-positive cells was observed in the liver of ATG-MO morphants at 3dpf. Data was obtained by counting PH3-positive cells versus total cells in a specific organ (e.g. liver) in sections from at least 3 st-MO embryos and 3 ATG-MO morphants at each stage, respectively (Table S1).

The small liver in leg1-MO^ATG^ morphants could be resulted from restricted cell cycle progression or increased apoptosis or both. To find out the cellular mechanism for the small liver phenotype, we first performed immunostaining analysis using an anti-phosphorylated Histone 3 (PH3) antibody on leg1-MO^ATG^ morphant and WT control embryos, respectively. Results showed that leg1-MO^ATG^ morphant at 38 hpf had significant less PH3-positive cells (22 PH3-positive cells out of 846 total cells counted from sections from 4 embryos, account for 2.6%) than that in the WT control (63 PH3-positive cells out of 923 cells counted from sections from 4 embryos, account for 6.8%) (Fig. 7A,B,E; Table S1). Counting of PH3-positive cells in the neural tube in the same sections revealed no significant differences between leg1-MO^ATG^ morphant (215 out of 9572 cells counted, accounting for 2.2%) and WT (178 out of 8535 cells counted, accounting for 2.1%) (Fig. 7A,B,E; Table S1). This data suggests that depletion of Leg1 results in cell cycle arrest specifically in the liver primordium but not in the neural tube. Interestingly, at 3 dpf, while the morphant liver is greatly reduced in size the ratio of PH3-positive cells (36 out of 897 cells counted from sections from 3 embryos, accounting for 4%) was not much different from that in WT (81 out of 2734 cells counted from sections from 3 embryos, account for 3%) (Fig. 7C–E). Next, we performed TUNEL assay to compare apoptotic activities in the liver of leg1-MO^ATG^ morphant and WT embryos at 3 dpf. As expected, no apoptotic cells (out of 2869 liver cells counted) were identified in the WT liver. To our surprise, no apoptotic cells (out of 679 liver cells counted) were identified in sections from three leg1-MO^ATG^ embryos at the liver site either. Therefore, the small liver phenotype in leg1-MO^ATG^ morphant is caused due to cell cycle arrest rather than due to cell apoptosis.

### Leg1a and Leg1b are novel secretory proteins

**Figure 8:**
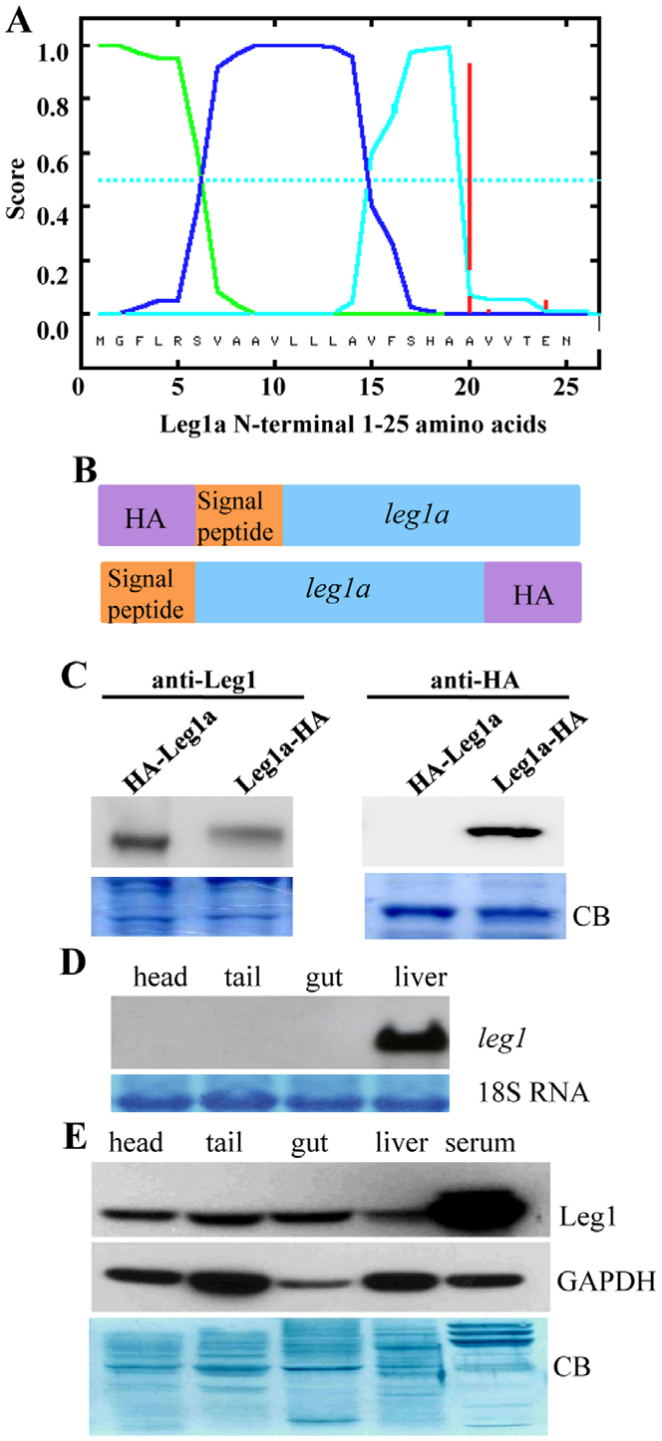
Leg1a is a secretory protein. (A) The first N-terminal 25 amino acids of Leg1a were shown on the X-axis. Y-axis represents probabilities. Green line: positively charged N-terminal end region; blue line: hydrophobic core sequence containing 12 uncharged amino acids; cyan line: recognition region by signal peptidase; red line: signal peptide cleavage site. (B) Diagram showing construction of *HA-leg1a* and *leg1a-HA* plasmids. (C) Western blotting detecting Leg1 proteins by anti-Leg1 monoclonal antibody (left panel) and detecting Leg1-HA fusion protein by anti-HA antibody (right panel). (D) Northern blotting detecting total *leg1* transcripts (*leg1a*+*leg1b*) in different tissues from adult fish. 18S RNA staining was used as the loading control. (E) Western blotting detecting total Leg1 (Leg1a+Leg1b) in different tissues from adult fish. GAPDH and Coomassie blue staining (CB) were used as the loading controls.

Analysis of Leg1a and Leg1b peptide sequences identified a signal peptide at their N-termini (Fig. 8A), suggesting that Leg1a and Leg1b are possibly secretory proteins. To determine whether Leg1a is indeed a secretory protein, we cloned *leg1a* coding sequence in-frame to the HA tag either before or after the tag into expression vector pCS2+ (constructs *leg1a-HA* and *HA-leg1a*) (Fig. 8B). *leg1a-HA* or *HA-leg1a* mRNA was respectively injected into embryos at one-cell stage. The scenario is that if Leg1 is secretory protein, the N-terminal tagged HA tag will be cleaved together with the signal peptide and the matured Leg1 would fail to be detected by anti-HA antibody. Conversely, if Leg1 is not a secretory protein, the anti-HA antibody must recognize both forms of Leg1 fusion proteins. Total proteins were extracted from injected embryos at 8hpf and subjected to western blotting analysis using monoclonal antibodies recognizing either HA tag or Leg1 protein. Results showed that the anti-Leg1 antibody detected Leg1a in both samples prepared from *HA-leg1a* and *leg1a-HA* mRNA injected embryos, respectively (Fig. 8C). Furthermore, Leg1 protein detected by anti-Leg1 antibody in the *leg1a-HA* mRNA injected embryos is higher in molecular weight than that that in the *HA-leg1a* mRNA injected embryos (Fig. 8C). On the other hand, the anti-HA antibody could only detect the HA-tagged protein in the *leg1a-HA* mRNA injected embryos (Fig. 8C). The results obtained suggest that Leg1a is secretory protein.

Next, we asked whether Leg1a and Leg1b are extracellular protein. In the adult fish, total *leg1* transcripts are detected mainly in the liver and are almost undetectable in the head, tail, and trunk (Fig. 8D). However, analysis of total Leg1 proteins via western blotting using anti-Leg1 antibody showed that total Leg1 were detected in these tissues in addition to the liver (Fig. 8E). Furthermore, total Leg1 levels in head, tail and trunk were comparable to that in the liver (Fig. 8E). Most strikingly, serum contained the highest level of total Leg1 (approximately three times higher) among all tissues tested (Fig. 8E). Therefore, Leg1 is a novel secretory protein produced by the liver.

### N-terminal signal peptide is essential for Leg1 function

**Figure 9:**
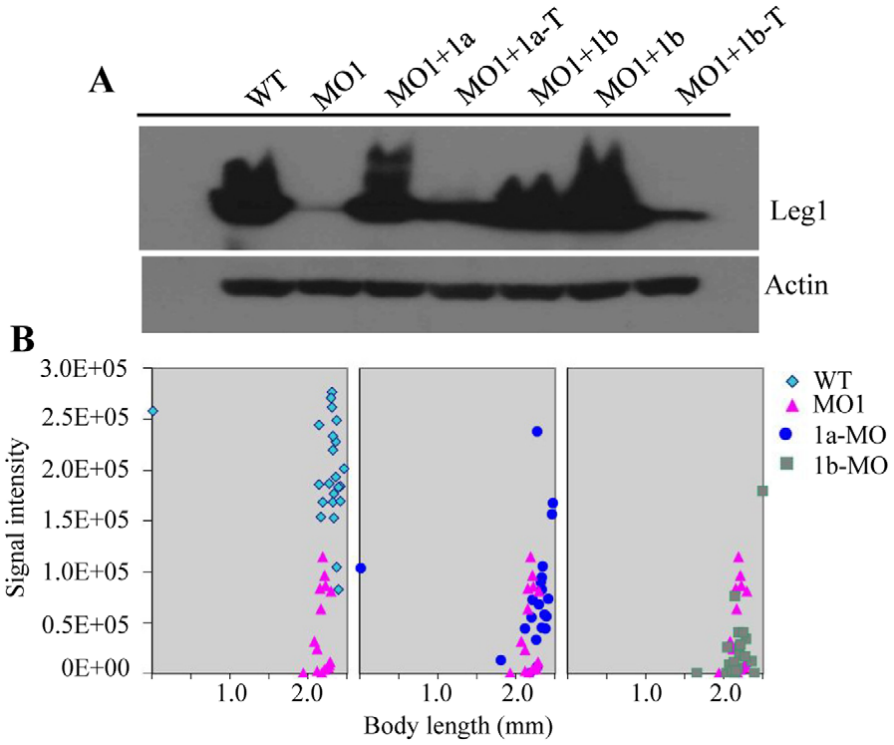
N-terminal signal peptide is essential for Leg1 function. (A) Western analysis of Leg1 protein expression in WT, leg1-MO^ATG^ morpahnt (MO-1), leg1-MO^ATG^ co-injected with *leg1a* (1a) (MO-1+1a lane) or *leg1b* (1b) (MO-1+1b lane) mRNA, with N-terminal signal peptide coding sequence truncated *leg1a* (1a-T) (MO-1+1a-T lane) or *leg1b* (1b-T) (MO-1+1b-T lane) mRNA. (B) Statistical analysis of liver size based on WISH signal intensity of *fabp10a* in each individual embryo from different treatment as stated. 20–23 embryos were used for statistical analysis in each case except for MO-1 (15 embryos were used).

Considering that Leg1 is a secretory protein, one intriguing question to ask is whether the N-terminal signaling peptide is necessary for Leg1's function. To address this question, we designed a construct which is deleted of the N-terminal signaling peptide. This construct is used for generating mRNA in vitro and such mRNA was co-injected with leg1-MO^ATG^ into embryos at one-cell stage (Fig. 9A). Examination of embryos four days post-injection with the *fabp10a* probe showed that the N-terminal signaling peptide truncated Leg1 failed to rescue the small liver phenotype caused by leg1-MO^ATG^ (Fig. 9B). This data suggests that the N-terminal signal peptide is essential for Leg1 function.

## Discussion

We chose *leg1* in this work mainly for two reasons: firstly, Leg1 is a novel protein whose function has never been reported in previous studies except for our preliminary data showing that Leg1 might be involved in zebrafish liver development [3]. Secondly, *leg1* expression is enriched in both adult and embryonic liver, we are intrigued to find out if Leg1 plays dual functions at these two developmental stages in zebrafish liver. In this report, we focused mainly on characterization of Leg1 protein and its role in early liver development.

We found that zebrafish has two closely related *leg1* homologs, namely *leg1a* and *leg1b*. *leg1a* and *leg1b* share high homology in their coding sequences, however, are divergent in their 5′-UTR sequences. Further studies showed that *leg1a* is predominantly expressed than is *leg1b* in both embryos and adult liver, demonstrating their expression is differentially regulated. Sequence alignment analysis showed that the 600 bp proximal promoter sequences (promoter sequence directly upstream of the transcription start site) share little homology between *leg1a* and *leg1b* although they do have two conserved regions in their distant promoter regions. This observation might explain why *leg1a* is a prominent form during embryogenesis whilst both *leg1a* and *leg1b* are highly expressed in the adult liver. Then, the intriguing question to ask is whether, during evolution, nature has assigned one homologue (i.e *leg1a*) to function in developmental process and the other (ie. *leg1b*) to be responsive to physiological or pathological stresses. Future effort is worth to be made to find out how this differential expression is achieved *in vivo*.

We previously showed that knockdown total Leg1 (Leg1a + Leg1b) by leg1^ATG^-MO conferred a small liver phenotype. In this report, we proved that the small liver phenotype was caused duo to cell cycle arrest rather than due to cell apoptosis. Apparently, our future work will need to elucidate why depletion of Leg1 specifically impairs the cell cycle process but not cell apoptosis. Upon discovery of *leg1a* and *leg1b* two homologs, we used highly potent leg1a-specific (leg1a-MO) and leg1b-specific (leg1b-MO) morpholinos to study their individual roles in liver development. Our results showed that knockdown of Leg1a depleted most of total Leg1 protein causing a more severe small liver phenotype whereas knockdown of Leg1b moderately lowered total Leg1 protein causing a less severe small liver phenotype. Interestingly, *leg1a* or *leg1b* mRNA injection alone partially rescued the small liver phenotype of leg1-MO^ATG^ morphants to similar extents, and was obviously less efficient than did the *leg1a* and *leg1b* mRNA co-injection. Taken together, these results suggest that Leg1a and Leg1b appear to play partially redundant roles in liver development. However, the best way to clarify the role of Leg1a and Leg1b in liver development is to get null mutations for each individual gene which, unfortunately, is not feasible at this moment.

At the transcriptional level, in the adult fish, total *leg1* transcripts were detected mainly in the liver and were almost undetectable in the head, tail, and trunk. At the translational level, in contrast, similar levels of Leg1 protein were detected in the head, gut and tail as that in the liver while Leg1 level is the highest in the serum. Based on this observation we reckoned that Leg1 is likely to be a novel liver-produced secretory protein. Protein domain analysis identified a putative signal peptide in Leg1. We confirmed this prediction by analyzing the translational product of injected *leg1* tagged with HA-tag at either the N-terminal or C-terminal of Leg1. Results showed that the HA-tag was cleaved off from Leg1 when it was tagged at the N-terminal of Leg1 whilst the HA-tag was still fused with Leg1 when being tagged at the C-terminal of Leg1. The nature of Leg1 to be a secreted protein might explain why organogenesis of the exocrine pancreas and intestine are also affected in the leg1-MO^ATG^ morphants if considering the hypothesis that Leg1 is a novel secreted growth regulator. In fact, our data showing that the N-terminal signal peptide is essential for Leg1 function provides a strong evidence to support this hypothesis.

In summary, we have here presented data to demonstrate that Leg1 is a novel liver-produced secretory protein that is essential for digestive organ development in zebrafish. However, many keys questions remain to be addressed in the future. For example, what is the biochemical function of Leg1? Is Leg1 a secreted enzyme, or a growth factor, or a carrier? Will Leg1 form a complex with other protein(s) and how does Leg1 exert its function on liver development? Future work to address these questions will help to unravel the biological functions of Leg1.

## Supporting Information

Figure S1 **Alignment of** ***leg1a*** **and** ***leg1b*** **full length cDNA sequences.** Translation start codon ATG and stop codon TGA of both genes are boxed in red.
(JPG)

Figure S2 **Alignment of** ***leg1a*** **and** ***leg1b*** **promoter sequences.** 3 kb of *leg1a* and *leg1b* genomic DNA sequences upstream of their respective transcription start sites (letter in lower case) were retrieved from Zv8/danRer6 assembled in UCSC Genome Broswer (http://genome.ucsc.edu/cgi-bin/hgGateway), respectively. For *leg1a*, the 3 kb is from the region of chr20:1,478,061–1,481,060, and for *leg1b*, chr20:1,463,371–1,466,370. Alignment was performed using Ebi Tool needle (http://www.ebi.ac.uk/Tools/psa/emboss_needle/nucleotide.html). Parameters were set as the following: Matrix: EDNAFULL, # Gap_penalty: 50.0, Extend_penalty: 0.5. Alignment shows that there are two conserved regions in *leg1a* and *leg1b* promoter (Region I, in red; Region II, in purple). However, the rest of sequence, especially the 600 bp proximal promoter sequence, is highly divergent between these two genes.
(JPG)

Table S1 **Statistical data for immunostaining of PH3.**
(DOC)
