# Supplementary figures and images for: liver-enriched gene 1a and 1b Encode Novel Secretory Proteins Essential for Normal Liver Development in Zebrafish
Source: PLoS One. 2011 Aug 9;6(8):e22910. doi: 10.1371/journal.pone.0022910 (PMC3153479; doi:10.1371/journal.pone.0022910)

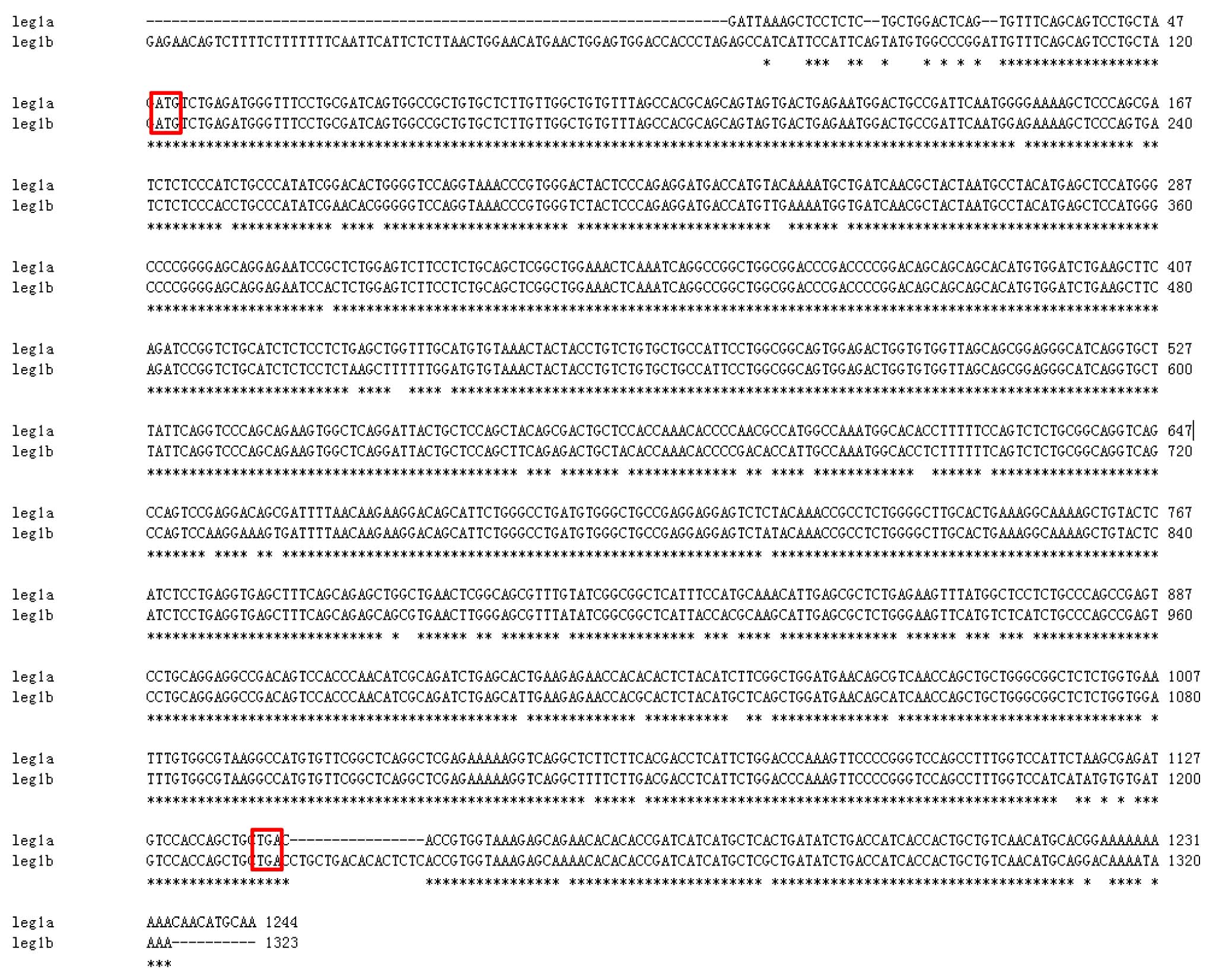

Supplement: Figure S1 — Alignment of leg1a and leg1b full length cDNA sequences. Translation start codon ATG and stop codon TGA of both genes are boxed in red. (JPG) [file pone.0022910.s001.jpg]

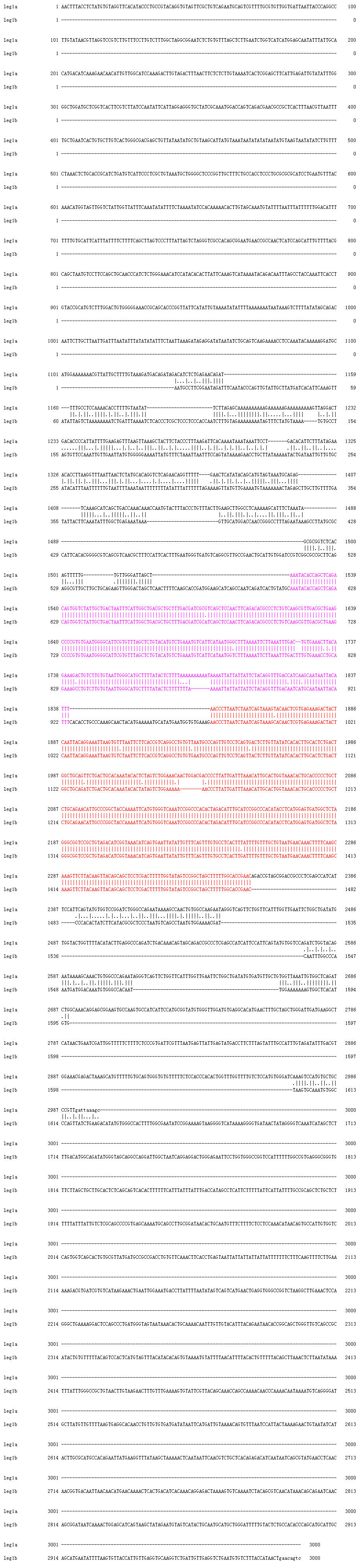

Supplement: Figure S2 — Alignment of leg1a and leg1b promoter sequences. 3 kb of leg1a and leg1b genomic DNA sequences upstream of their respective transcription start sites (letter in lower case) were retrieved from Zv8/danRer6 assembled in UCSC Genome Broswer (http://genome.ucsc.edu/cgi-bin/hgGateway), respectively. For leg1a, the 3 kb is from the region of chr20:1,478,061–1,481,060, and for leg1b, chr20:1,463,371–1,466,370. Alignment was performed using Ebi Tool needle (http://www.ebi.ac.uk/Tools/psa/emboss_needle/nucleotide.html). Parameters were set as the following: Matrix: EDNAFULL, # Gap_penalty: 50.0, Extend_penalty: 0.5. Alignment shows that there are two conserved regions in leg1a and leg1b promoter (Region I, in red; Region II, in purple). However, the rest of sequence, especially the 600 bp proximal promoter sequence, is highly divergent between these two genes. (JPG) [file pone.0022910.s002.jpg]
