# Supplementary material for: liver-enriched gene 1a and 1b Encode Novel Secretory Proteins Essential for Normal Liver Development in Zebrafish
Source: PLoS One. 2011 Aug 9;6(8):e22910. doi: 10.1371/journal.pone.0022910 (PMC3153479; doi:10.1371/journal.pone.0022910)
Supplement: Table S1 — Statistical data for immunostaining of PH3. (DOC) [file pone.0022910.s003.doc]

**SUPPORITNG INFORMATION**

**Table S1. Statistical data for immunostaining of PH3**

| Stage | Sample | | Fish1* | Fish2* | Fish3* | Fish4* | Average |
| --- | --- | --- | --- | --- | --- | --- | --- |
| 38hpf | st-MO | Neural tube | 42/2342 (1.79) | 57/2359 (2.42) | 62/2350 (2.64) | 54/2521 (2.14) | 2.25 |
|  |  | Liver | 15/156 (9.62) | 17/272 (6.25) | 15/235 (6.38) | 16/260 (6.15) | 7.10 |
|  | ATG-MO | Neural tube | 44/2177 (2.02) | 43/2015 (2.13) | 54/2457 (2.20) | 37/1886 (1.96) | 2.08 |
|  |  | Liver | 9/211 (4.27) | 3/222 (1.35) | 4/173 (2.31) | 6/240 (2.50) | 2.61 |
| 3dpf | st-MO | Neural tube | 9/3356 (0.27) | 10/4873 (0.21) | 12/4622 (0.26) |  | 0.25 |
|  |  | Liver | 12/620 (1.94) | 29/978 (2.97) | 40/1136 (3.52) |  | 2.81 |
|  | ATG-MO | Neural tube | 25/4498 (0.56) | 12/3720 (0.37) | 9/3445 (0.26) |  | 0.40 |
|  |  | Liver | 9/191 (4.71) | 15/394 (3.81) | 12/312 (3.85) |  | 4.12 |

*Data were presented with PH3-positive cells over total cells counted. The percentage of PH3-positive cells for each fish was shown in the bracket. Average percentage was obtained based on the percentage for each individual fish.
